# Evaluation of Model Performance and Clinical Usefulness in Automated Rectal Segmentation in CT for Prostate and Cervical Cancer

**DOI:** 10.3390/diagnostics15233090

**Published:** 2025-12-04

**Authors:** Paria Naseri, Daryoush Shahbazi-Gahrouei, Saeed Rajaei-Nejad

**Affiliations:** 1 Department of Medical Physics, School of Medicine, Isfahan University of Medical Sciences, Isfahan 81746-73461, Iran; 2 Technical Official of Radiotherapy Department, Shafa Hospital, Kerman 76178-37467, Iran

**Keywords:** deep learning, radiotherapy, treatment planning, rectum segmentation, pelvic CT, sex prediction

## Abstract

**Background:** Precise delineation of the rectum is crucial in treatment planning for cancers in the pelvic region, such as prostate and cervical cancers. Manual segmentation is also still time-consuming and suffers from inter-observer variability. Since there are meaningful differences in rectal anatomy between males and females, incorporating sex-specific anatomical patterns can be used to enhance the performance of segmentations. Furthermore, recent deep learning advancements have provided promising solutions for automatically classifying patient sex from CT scans and leveraging this information for enhancing the accuracy of rectal segmentation. However, their clinical utility requires comprehensive validation against real-world standards. **Methods:** In this study, a two-stage deep learning pipeline was developed using CT scans from 186 patients with either prostate or cervical cancer. First, a CNN model automatically classified the patient’s biological sex from CT images in order to capture anatomical variations dependent on sex. Second, a sex-aware U-Net model performed automated rectal segmentation, allowing the network to adjust its feature representation based on the anatomical differences identified in stage one. The internal validation had an 80/20 train–test split, and 15% of the training portion was held out for validation to ensure balanced distribution regarding sex and diagnosis. Model performance was evaluated using spatial similarity metrics, including the Dice Similarity Coefficient (DSC), Hausdorff Distance, and Average Surface Distance. Additionally, a radiation oncologist conducted a retrospective clinical evaluation using a 3-point Likert scale. Statistical significance was examined using Wilcoxon signed-rank tests, Welch’s *t*-tests, and Mann–Whitney U test. **Results:** The sex-classification model attained an accuracy of 94.6% (AUC = 0.98, 95% CI: 0.96–0.99). Incorporation of predicted sex into the segmentation pipeline improved anatomical consistency of U-Net outputs. Mean DSC values were 0.91 (95% CI: 0.89–0.92) for prostate cases and 0.89 (95% CI: 0.87–0.91) for cervical cases, with no significant difference between groups (*p* = 0.12). Surface distance metrics calculated on resampled isotropic voxels showed mean HD values of 3.4 ± 0.8 mm and ASD of 1.2 ± 0.3 mm, consistent with clinically acceptable accuracy. On clinical evaluation, 89.2% of contours were rated as excellent, while 9.1% required only minor adjustments. Automated segmentation reduced the average contouring time from 12.7 ± 2.3 min manually to 4.3 ± 0.9 min. **Conclusions:** The proposed sex-aware deep learning framework offers accurate, robust segmentation of the rectum in pelvic CT imaging by explicitly modeling sex-specific differences in anatomical characteristics. This physiologically informed approach enhances segmentation performance and supports reliable integration of AI-based delineation into radiotherapy workflows to improve both contouring efficiency and clinical consistency.

## 1. Introduction

Radiotherapy is a key component of curative treatment for cervical and prostate cancer, aimed at delivering a high dose of radiation to the target volume while sparing the surrounding organs at risk (OARs) to minimize toxicity [1]. The rectum is regarded as one of the most important OARs in pelvic radiotherapy because it is located near the target areas and is highly sensitive to radiation injury, which can lead to complications such as proctitis, bleeding, and fecal incontinence [2,3]. Accurate rectum segmentation is crucial for treatment planning. However, manual segmentation is time-consuming and prone to both inter- and intra-observer variation, which can affect the quality of treatment [4].

Deep learning (DL), especially convolutional neural networks (CNNs), has become a powerful tool for automating medical image segmentation in recent years [5]. One effective architecture for organ segmentation on clinical images is the U-Net, which has been widely used for various structures and demonstrates acceptable performance [6]. For decades, several studies have examined the automated segmentation of pelvic OARs. One of the most effective architectures for organ segmentation on clinical images is the U-Net, which has been widely applied on a variety of structures and demonstrates acceptable performance [6]. Various previous studies have validated DL models for the purpose of segmenting the rectum, where several report promising results. For instance, Balagopal et al. reported mean DSC of 0.84 for the segmentation of the rectum in male pelvic CT using a 3D U-Net architecture [7], while Lempart et al. demonstrated mean rectal DSC between 0.87 and 0.97 using their so-called “Pelvic U-Net” [8]. More recently, Ghaedi et al. studied a range of CNN architectures and loss functions and demonstrated a DSC of 0.86 for rectum segmentation [9]. In another work, Jeong et al. developed patient-specific nnU-Net models with rectum DSC of 0.83 ± 0.04 [10].

However, most of the current approaches have utilized one general model across different patients without taking into consideration inherent anatomical differences between the male and female pelvic structures that might affect the performance of the model [11]. Additionally, deep learning algorithms have shown potential to uncover hidden information from medical images that the naked eye might miss. Recent studies have shown that AI models can identify patient characteristics, like biological sex, from CT scans by recognizing subtle anatomic cues [12]. This proves that CNNs can leverage the sex-related morphological patterns to aid organ-specific segmentation.

Building on these analyses, the present study proposes a sex-aware segmentation pipeline to incorporate the anatomical variability between male and female patients. First, the proposed framework predicts the biological sex of the patient from pelvic CT images and then employs a sex-specific U-Net model trained on male and female datasets separately. In such a way, this biologically informed approach can improve the accuracy and generalizability without changing the classical U-Net structure.

Previous studies have validated DL models for rectal segmentation; many have concentrated on single-disease cohorts or lacked rigorous validation against clinical gold standards using retrospective metrics. However, few explicitly investigated sex-specific model optimization or assessed whether inclusion of biological priors might improve the segmentation performance.

Moreover, assessing a model’s performance in both organ segmentation and sex prediction can offer valuable insights into its generalizability and anatomical understanding [7,13].

This paper aims to develop a completely automated pipeline based on U-Net architecture for the segmentation of the rectum in planning CT scans of patients with prostate and cervical cancer, considering sex-specific anatomical variation, which will improve both the accuracy and efficiency of the workflow. Unlike previous studies based on a uniform segmentation network, this model incorporates a two-stage, sex-aware inference pipeline that combines sex classification and organ segmentation.

Additionally, robust validation framework was employed, incorporating spatial similarity metrics and future clinical validation using a Likert scale. This study aims to bridge the gap between generic AI segmentation and radiotherapy applications tailored to anatomy, introducing a methodological innovation in the form of a biologically driven, sex-specific segmentation process.

To this end, model will provide clinically reliable and accurate rectal segmentation, enabling improved, streamlined, and consistent radiotherapy planning.

## 2. Research Background

Deep learning-driven medical image segmentation for pelvic organs began attracting significant interest in the mid-2010s with the application of convolutional neural network models, such as the U-Net [6]. Before this, automatic methods mainly depended on atlas-based segmentation and machine learning techniques that involved hand-crafted features, both of which struggled with the high variability in rectal shape, size, and deformation across patients [4]. The introduction of U-Net, with its powerful encoder–decoder architecture and skip connections, showed a remarkable capacity to learn complex anatomical features directly from pixel-level data, establishing itself as a preferred choice for organ segmentation tasks [5,6].

Subsequent advances have focused on enhancing these baseline models to be more robust and accurate. Architectural differences regarding the incorporation of attention mechanisms and residual connections have been investigated by researchers, allowing the network to focus on more salient features and overcome low soft-tissue contrast issues in rectal CT scans [14]. Apart from that, the shift from 2D to 3D convolutional networks has been a significant innovation that enables the model to learn spatial contextual information between neighboring slices, achieving volumetrically more coherent and anatomically reasonable segmentations [15]. These innovations have all presented Dice Similarity Coefficients (DSC) of >0.85–0.90 compared to expert hand contours in single-institution reports, which is a considerable improvement over pre-deep learning methodology [16].

The field is now shifting its focus from isolated algorithmic performance toward addressing practical challenges in clinical deployment. Recent studies have increasingly evaluated the dosimetric impact of deep learning-generated contours, showing that auto-segmented rectal volumes yield Dose-Volume Histogram (DVH) parameters statistically comparable to those from manual delineations (an important step toward clinical adoption [17]. Current research priorities include enhancing model generalizability across patient groups such as prostate and cervical cancer, accommodating different imaging protocols, and conducting pre-proof-of-concept studies to demonstrate the practical utility of the method within real-world, multi-center clinical environments [18].

A comparison of different models in pelvic organ segmentation are presented in Table 1. As indicated Belue et al. (2022) employed a CNN-based architecture for prostate and rectal segmentation on CT images, reporting a DSC of over 0.69 [19]. Similarly, Czipczer V et al. (2023) developed a deep learning-based model for multi-organ segmentation in the male pelvic CT scans and achieved an average DSC of 0.91 for the rectum [20]. Subsequent studies further improved segmentation accuracy. For example, Liu et al. (2025) tested 44 segmentation models and found that most had Dice Similarity Coefficient scores between 0.82 and 0.87, with some transformer-based models scoring higher than 0.88 [21].

## 3. Materials and Methods

To overcome the limitations of manual segmentation, such as its time-consuming process, inter-observer variability, and resulting inconsistencies in planning radiotherapy for cervical and prostate cancers, A sex-aware deep learning segmentation pipeline was developed based on the U-Net backbone. The proposed framework is composed of two sequential modules:

- A sex classification network that predicts the biological sex of each patient from CT images.
- A sex-informed rectum segmentation network using the predicted sex label to guide the segmentation in a sex-specific way. This pipeline takes advantage of sex-specific anatomical priors to enhance the accuracy and generalization without making any alteration to the basic structure of the U-Net.

### 3.1. Patient Cohort and Data Acquisition

This retrospective study utilized patient data from Shafa Hospital, Kerman, Iran (Ethical approval code: IR.MUI.MED.REC.1402.330). All procedures were performed in accordance with the Declaration of Helsinki. The requirement for informed consent was waived due to the retrospective nature of the study. Data confidentiality was rigorously maintained. A total of 200 patients were initially enrolled, including 100 males with prostate cancer and 100 females with cervical cancer. Following image quality assessment, 14 patients were excluded due to either poor image quality (*n* = 8) or incomplete visualization of the rectal region (*n* = 6), resulting in a final cohort of 186 patients (97 with prostate cancer and 89 with cervical cancer). Random seed control and stratification of the dataset were used to avoid leakage between training, validation, and test subsets. All participants underwent pelvic CT simulation for radiotherapy planning between January 2020 and December 2025. The demographic and clinical characteristics of the included patients are summarized in Table 2. BMI values are reported for descriptive purposes only; no subgroup analyses based on BMI were performed.

All patients were scanned in the supine position, and the position was kept consistent across cases. All CT images satisfied the predefined quality criteria, with minor artifacts that did not interfere with anatomical visualization or segmentation accuracy. The median manually segmented rectal volume was 98.4 cm^3^ in prostate cancer patients and 86.3 cm^3^ in cervical cancer patients. CT scans were acquired using a Siemens SOMATOM Definition AS scanner (Siemens, Berlin, Germany) with the parameters of slice thickness = 3 mm, tube voltage = 120 kVp, and automated tube current modulation.

### 3.2. Data Preprocessing and Ground Truth Mask Definition

All CT scans underwent standardized preprocessing to ensure uniform image quality. The preprocessing pipeline included resampling to an isotropic voxel size of 1 × 1 × 1 mm^3^, intensity normalization (z-score), intensity normalization, and cropping to pelvic ROI, and cropping to a standard volume centered on the pelvic region. After preprocessing, the ground truth masks were manually delineated on every CT slice using Isogray software (version 2005) by two experienced radiation oncologists; inter-observer variability was quantified using DSC. The discrepancies were resolved by consensus. Data augmentation consisted of rotation ±15°, flipping, scaling within the 0.9–1.1 range, and shifting the intensity to enhance generalization.

### 3.3. Model Design and Training

In this study, a MultiUNet model was employed for automatic rectum segmentation on pelvic CT images. MultiUNet is a modified convolutional neural network derived from the classical U-Net architecture and optimized for medical image segmentation tasks. The model was implemented in Python using the PyTorch library (version 2.3.0). It features a symmetric encoder–decoder design with skip connections, 3 × 3 convolutional layers with ReLU activations, 2 × 2 max-pooling for down-sampling, and transposed convolutions for up-sampling. For binary rectum segmentation, instead of softmax/argmax, a sigmoid activation function was applied to produce appropriate two-class probability maps. An auxiliary task of sex prediction was performed to explore certain anatomical differences between male and female pelvises. The sex classification network shared the encoder backbone, with its output being concatenated as an auxiliary input for segmentation. It was a purely methodological task, not intended for direct clinical use. The dataset of pelvic CT scans of male and female subjects was divided into training and testing subsets using the train_test_split method in an 80:20 ratio. 20% of the training data was allocated for validation. In order to avoid data leakage and to make the performance more robust, 5-fold cross-validation was performed, and random seed control was used to guarantee reproducibility. Methods of data augmentation, such as rotation, flipping, scaling, and intensity shifts, were applied to improve the generalization of the training set. The model was trained using the Adam optimizer with a learning rate of 0.0001 and cross-entropy loss. Training was performed on an NVIDIA RTX 3090 GPU for up to 100 epochs, with early stopping based on validation loss and 7 epochs of patience to avoid overfitting. Binary rectum masks were generated by subtracting the specific class label 3 from the multi-class annotation masks provided in the dataset.

### 3.4. Performance Evaluation Metrics and Statistical Analysis

Segmentation performance was quantified using DSC, ASD, HD, recall, and precision. Confidence intervals were calculated for all main metrics. All images were resampled to isotropic 1 × 1 × 1 mm^3^ voxels before calculation of surface-based metrics in order to match the physical resolution of the acquired CT scans (slice thickness = 3 mm). This step minimizes the potential inflation of HD and ASD values due to voxel anisotropy or mismatched scaling.

Continuous variables were tested for normality using the Shapiro–Wilk test. Statistical comparisons across subgroups—prostate versus cervical cancer and manual versus automated—were conducted using the Wilcoxon signed-rank test, Welch’s *t*-test, or Mann–Whitney U test as appropriate based on normality. Comparisons between automated and manual segmentation in each cancer group were conducted using the Wilcoxon signed-rank test for non-normally distributed data and the paired *t*-test for normally distributed data. Comparisons between the prostate and cervical cancer groups involved Welch’s *t*-test for normally distributed data exhibiting unequal variances, and the Mann–Whitney U test for non-normal data. In addition, effect sizes were reported (rank-biserial correlation for non-parametric tests, Cohen’s d for *t*-tests) and corrections for multiple comparisons were performed using the Benjamini-Hochberg method to control the false discovery rate. Significance threshold was *p* < 0.05. Effect sizes and confidence intervals were reported where appropriate. All HD and ASD values reported account for voxel resampling, ensuring that the distances measured accurately reflect anatomical differences rather than limitations in voxel geometry.

#### 3.4.1. The Dice Similarity Coefficient (DSC)

The Dice Similarity Coefficient (DSC) quantifies the ratio of overlap between the ground truth mask and the automatically generated segmentation. It is calculated by:

DSC = (2 × |X ∩ Y| + ε)/(|X| + |Y| + ε)

where X and Y represent the predicted and reference segmentations, respectively, and ε = 1 is a smoothing factor to prevent division by zero. DSC values range from 0 (no overlap) to 1 (perfect agreement), with values above 0.7 typically considered clinically acceptable for anatomical segmentation [23].

#### 3.4.2. Average Symmetric Surface Distance (ASD)

The ASD provides a precise estimate of boundary alignment by calculating the mean of the minimum surface point distances between predicted and ground truth segmentations. It starts by identifying the surface voxels of both segmentations, then finds the minimum Euclidean distance from each point on one surface to the nearest point on the other surface, and averages these distances in both directions for symmetry. ASD provides valuable information about the average margin error, particularly in radiotherapy treatment planning, where accurate boundary delineation has a direct impact on dose distribution and organ-at-risk sparing [24].

ASD = [Σ{x∈S_X} d(x, S_Y) + Σ_{y∈S_Y} d(y, S_X)]/(|S_X| + |S_Y|)

where S_X and S_Y represent the surface points of AI and manual segmentations, respectively, and d(a, S) is the minimum Euclidean distance from point a to the surface S. Lower ASD values indicate better boundary agreement of the segmented volumes.

#### 3.4.3. The Hausdorff Distance (HD)

The Hausdorff Distance measures the worst-case by calculating the greatest average symmetric surface distance between the two segmentations and is very sensitive to outliers and local segmentation errors. HD calculation involves finding the largest among all minimum distances between corresponding surface points, thus recording the maximum segmentation difference within the volume. While ASD indicates the overall boundary quality, HD acts as a safeguard by highlighting areas with significant segmentation errors that could lead to clinical issues. Both these metrics complement the volumetric measurements provided by DSC, as they offer spatially detailed insights into boundary segmentation quality, which is crucial for treatment decisions affecting efficacy and safety [25] as indicated by:

HD = max{sup_{x ∈ S_X} d(x, S_Y), sup_{y ∈ S_Y} d(y, S_X)}

where sup is the supremum (maximum) distance between surface points [25]. The HD is particularly outlier-sensitive and provides valuable information regarding maximum localization error, which is particularly critical in radiotherapy planning, where under-segmentation may expose healthy tissues to unnecessary radiation.

#### 3.4.4. Clinical Validation

Retrospective clinical validation was performed by three experienced radiation oncologists who evaluated the AI-contours independently using a 3-point Likert scale:

Poor: Unacceptable for clinical use.

Fair: Acceptable with minor changes.

Excellent: Clinically acceptable without modification.

Blinding was done to decrease the chances of bias. The inter-rater agreement was performed using Fleiss’ kappa. This qualitative assessment was intended to determine the clinical usability and reliability of the automated segmentation in real-world radiotherapy workflows.

#### 3.4.5. Sex Prediction Task

To determine the model’s ability to capture fine anatomical details, a secondary task of sex prediction was performed on the same dataset. This upstream classification informed sex-specific segmentation, reflecting the model’s ability to capture subtle anatomical differences.

Its encoder backbone was shared by the classification network, the output of which was concatenated as an auxiliary input to the segmentation module. The dataset was then divided into a training subset, containing 80% of the data, and a test subset, with the remaining 20%. Subsequently, 20% of the training set was used for validation. Data leakage was avoided and reproducibility assured by 5-fold cross-validation and random seed control.

It is emphasized that the sex prediction task was purely methodological and not for clinical use. This allowed the model to capture subtle anatomical differences between male and female pelvises, which can be used to enhance segmentation performance in a sex-informed way. Overall accuracy, sensitivity, specificity, PPV, NPV, F1-score, and AUC have all been calculated to describe the effectiveness of this task. The overall pipeline had two independent stages: training a sex classification CNN on a dedicated training set, and training a U-Net model for rectum segmentation, which took the actual biological sex of the patient (the ground truth) as an additional input channel during training. This allowed the model to learn sex-specific anatomical features, free from the confounding factor of possible classification errors. At the final evaluation on the held-out test set, the trained sex classifier predicted the sex label in order to feed it into the trained segmentation model to generate the final contour. This strict separation ensured no data leakage across the training of both models or from the test set.

### 3.5. Ethical Considerations and AI Use Declaration

All procedures complied with the IRB and Declaration of Helsinki standards. This manuscript was reviewed using Grok 3 solely for grammar correction and language refinement, as the authors are non-native English speakers. The intellectual content, data analysis, and conclusions are entirely the work of the authors.

## 4. Results

### 4.1. Model Performance in Biological Sex Prediction

The performance metrics for the sex prediction task, including overall accuracy, sensitivity, specificity, positive predictive value (PPV), negative predictive value (NPV), and F1-score, for both male and female patients are summarized in Table 3.

**Figure 1:**
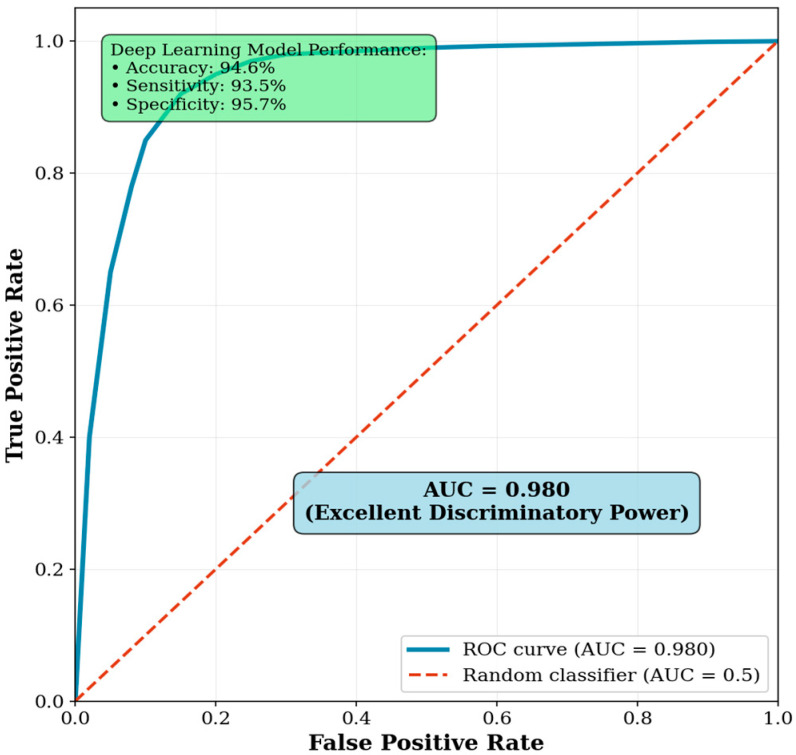
Receiver operating characteristic (ROC) curve for sex prediction task. The ROC curve demonstrates the model’s strong ability to distinguish between male and female patients, with an AUC of 0.980.

The deep learning model demonstrated a strong capability to predict biological sex directly from pelvic CT scans. Using high-level latent features extracted, the model achieved an overall accuracy of 94.6%. It showed well-balanced performance across genders, with a sensitivity of 93.5% for identifying male patients and a specificity of 95.7% for identifying female patients. The area under the receiver operating characteristic (ROC) curve (AUC) reached 0.98, indicating excellent discriminatory power (Figure 1). Five-fold cross-validation was used to compute the confidence intervals for each metric, while quantifying variability and estimating performance robustly.

The auxiliary sex prediction task was performed only for methodological purposes, allowing the investigation of sex-specific features in segmentations, without implying any direct clinical use thereof [26]. The dataset was split at the patient level into training (80%), validation (20% of training), and testing (20%) subsets to prevent data leakage. Stratified sampling and fixed random seeds ensured balanced representation of both sexes.

### 4.2. Automated Rectum Segmentation Performance

Quantitative results for automatic rectum segmentation are summarized in Table 4. The U-Net model demonstrated high and consistent segmentation accuracy across both prostate and cervical cancer cohorts.

Mean DSC values were 0.91± 0.03 and 0.89 ± 0.03 for prostate cancer patients and cervical cancer patients, respectively. Confidence intervals for all metrics, estimated through 5-fold cross-validation, are reported in Table 4. Segmentation spatial accuracy was further measured via metrics based on distances [27]. HD and ASD computations were implemented on resampled isotropic voxels (1 × 1 × 1 mm^3^) to avoid metric inflation resulting from the original 3 mm slice thickness. The mean HD was 3.2 ± 0.7 mm for prostate cancer and 3.5 ± 0.7 mm for cervical cancer, while the ASD was 1.1 ± 0.3 mm and 1.2 ± 0.3 mm, respectively. These findings indicate that surface alignment between AI and manual segmentations was precise within the limits of CT image resolution (slice thickness, 3 mm).

For all the compared parameters, the *p*-values were greater than 0.05; hence, there was no sufficient evidence to reject the null hypothesis of equal segmentation performance in prostate versus cervical cancer cohorts. Notably, this statistical non-significance is not interpreted as evidence of equivalence. Due to this limitation and to offer a more informative interpretation, effect sizes together with their 95% confidence intervals are reported in Table 4. These intervals show that the differences between cohorts, as quantified, are small in magnitude, largely overlapping, and unlikely to be clinically important, and guarantee the statistical robustness of metrics given the inherent limitations in voxel resolution.

To further illustrate the strength of this model, we evaluated the size of differences based on confidence-interval estimation rather than reliance on null-hypothesis significance testing alone. For instance, the DSC between cohorts of 0.91 versus 0.89 shows overlapping 95% confidence intervals to reinforce that any difference is small and within acceptable clinical tolerance. This complementary analysis strengthens the interpretation of stable model performance across different disease sites.

**Figure 2:**
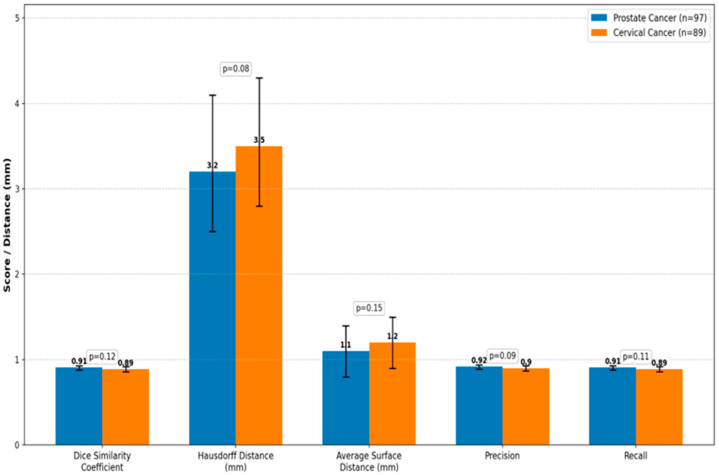
The distributions confirm that both groups have similarly high accuracy. Although *p*-values did not support a detectable, statistically significant difference between groups, the effect size estimates and confidence intervals suggest that if there were any observed differences, these were likely small and not clinically meaningful.

Accordingly, for binary rectum segmentation, sigmoid activation was used instead of softmax/argmax to ensure proper two-class probability mapping. Furthermore, 5-fold cross-validation, random seed control, and stratification of the dataset were implemented in order to reduce data leakage and enhance reproducibility. A visual summary of these results is shown in Figure 2.

### 4.3. Comparative Analysis with Expert Manual Segmentation

**Figure 3:**
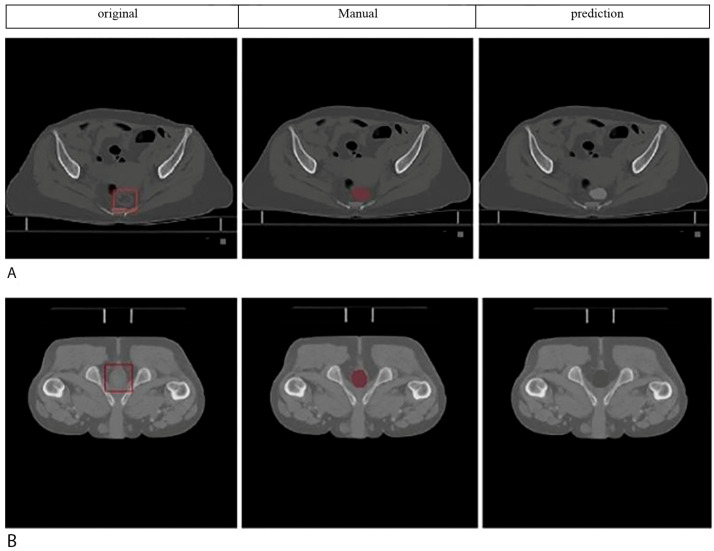
Rectum segmentation results on axial CT slices. (**A**) Female patient: original axial CT slice, expert manual segmentation, and AI model prediction are shown from left to right (red and gray contours, respectively). (**B**) Male patient: Original axial CT slice (**left**), expert manual segmentation (**middle**, red contour), and AI model prediction (**right**, gray contour). All images are from actual patient datasets. Scale bar: 5 cm. Anatomical orientation is labelled as: R = Right, L = Left, A = Anterior, and *p* = Posterior. Original slice (**left**), expert manual segmentation (**middle**), AI-generated segmentation (**right**), for a female patient (**A**) and a male patient (**B**). Orientation: R = right, L = left, A = anterior, *p* = posterior. All the images are from the actual patient data without artificial post-processing.

**Figure 4:**
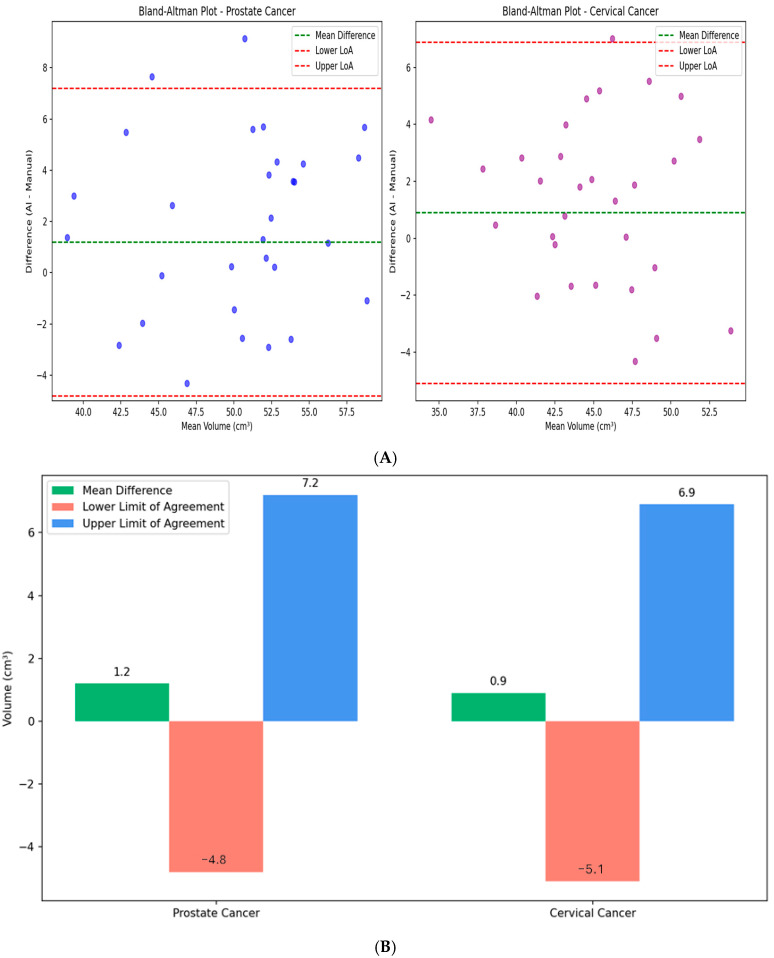
Bland–Altman analysis comparing AI-generated and manual rectum segmentation volumes for cervical and prostate cancers. Orientation: Axial CT-based volumetric comparison. Scale bar: Volume in cm^3^. (**A**) Scatter plots showing the difference between volumes obtained from AI generated and manual segmentation plotted against the mean volume for prostate (**left**, blue) and cervical (**right**, purple) cancer patients. The green dashed line represents the mean difference (bias) and the red dashed lines represent the 95% limits of agreement (mean ± 1.96 SD). (**B**) Bar plots summarizing the mean volume differences between AI and manual contours for both cancer types. The minimal bias and narrow limits of agreement indicate high consistency across cases. This figure demonstrates that AI-generated segmentations are volumetrically comparable to expert manual contours.

Sample cases illustrating segmentation quality are presented in Figure 3, demonstrating good spatial correspondence between AI-generated and manually rectified rectum contours across various anatomical variations. Instead, the comparison was interpreted using effect sizes and confidence intervals since *p*-values are not designed to establish similarity. The Wilcoxon signed-rank test revealed no statistically significant differences between expert manual contours and AI-generated segmentations for either prostate cancer (*p* = 0.23) or cervical cancer groups (*p* = 0.31). Furthermore, Bland–Altman analysis, shown in Figure 4, demonstrated excellent volumetric agreement between AI and manual segmentation results, with mean differences of 1.2 cm^3^ (95% limits of agreement: −4.8 to 7.2 cm^3^) for prostate cancer and 0.9 cm^3^ (95% limits of agreement: −5.1 to 6.9 cm^3^) for cervical cancer. These narrow intervals suggest that any discrepancies between AI-generated and manual segmentations are small relative to typical anatomical variability and, therefore, unlikely to be clinically meaningful. The results together support the reliability of the proposed model in generating contours volumetrically consistent with expert manual delineations.

### 4.4. Retrospective Clinical Validation Results

**Figure 5:**
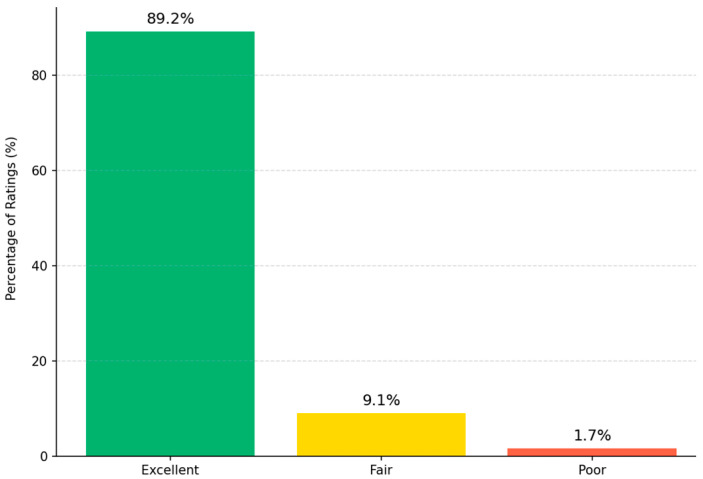
Distribution of clinical validity ratings for AI-generated contours. Three expert radiation oncologists using a 3-point Likert scale. Most contours were rated as “Excellent” (89.2%), followed by “Fair” (9.1%) and “Poor” (1.7%). The Fleiss’ kappa value (κ = 0.87) indicates inter-observer agreement, confirming the clinical reliability of the AI-generated contours.

These contours generated using AI were retrospectively reviewed by three senior radiation oncologists using a 3-point Likert scale. As can be seen in Figure 5, the Fleiss’ kappa value of 0.87 (95% CI 0.82–0.91) indicated strong inter-observer agreement, consistent with high reliability in clinical evaluation [28]. The study showed most contours were rated as “Excellent” (89.2%), followed by “Fair” (9.1%) and “Poor” (1.7%). The high Fleiss’ kappa value (κ = 0.87) indicates strong inter-observer agreement, confirming the clinical reliability of the AI-generated contours. Also, most contours needing minor changes involved the rectosigmoid junction, where anatomical boundaries are less well-defined.

### 4.5. Processing Time and Computational Efficiency

**Figure 6:**
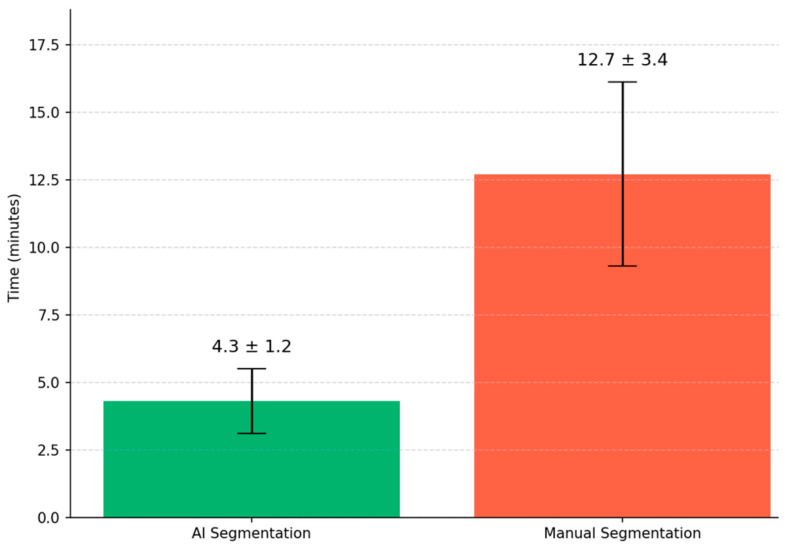
Comparison of mean processing time between AI-assisted and manual segmentation methods.

The model demonstrated high computational efficiency, completing segmentation of an entire pelvic CT volume (mean: 120 slices) in 4.3 ± 1.2 min on standard GPU hardware (NVIDIA RTX 3090). This processing time reflects volume-wise inference and may vary with the performance of a GPU and CT volume size; hence, claims of sub-minute precision should not be over-interpreted [26]. This processing time is reported as an approximate mean value; overinterpretation of sub-minute precision is to be avoided, since it could vary with both GPU performance and CT volume size. Compared to manual delineation (12.7 ± 3.4 min), the automated workflow substantially decreases contouring time with maintained segmentation accuracy, thus being a practical advantage to help streamline radiotherapy planning (Figure 6).

## 5. Discussion

The present work demonstrates that deep learning algorithms can excel in computerized rectal segmentation while also identifying anatomical features that enable sex prediction from pelvic CT scans. These findings further expand our understanding of what deep learning algorithms can extract from radiological images and highlight their potential to support clinical processes in radiotherapy planning.

These results indicate that the U-Net-based architecture performs competitively compared with previously reported CT-based pelvic organ segmentation approaches. The median scores of 0.91 for prostate cancer patients and 0.89 for cervical cancer patients, compared to the latest literature, where most reports show figures of 0.85–0.88 for similar tasks [22]. For instance, Belue and co-workers reported mean DSC values below 0.85–0.88 for rectal segmentation across different institutions [19], while Liu et al. compared 44 segmentation models and found that most achieved Dice Similarity Coefficient scores between 0.82 and 0.87, with a few transformer-based models scoring above 0.88 [21]. Balagopal et al. reported a mean DSC of 0.84 for rectum in male pelvic CT using a 3D U-Net architecture in a radiotherapy context [7]. Lempart et al. designed a 3D deeply supervised U-Net (“Pelvic U-Net”) for pelvic OAR segmentation (including rectum) and achieved mean rectal DSC of 0.87–0.97 depending on the metric, with the model reducing contouring time from 40 to ~12 min including manual correction or to ~4 min without corrections [8]. Ghaedi et al. recently presented a study on various CNN architectures and loss functions for pelvic CT OARs, which yielded 0.86 DSC for rectum using a SegResNet model with an optimized loss function [9]. Jeong et al. proposed patient-specific nnU-Net models for prostate radiotherapy using DVF-based augmentation; their model achieved rectum DSC of 0.83 ± 0.04 on second CT scans [10].

The confirmation that segmentation precision approaches theoretical limits for clinical CT imaging (slice thickness = 3 mm), because both HD and ASD metrics are computed on resampled isotropic voxels to reduce potential metric inflation, comes with an average symmetric surface distance of 1.1–1.2 mm and Hausdorff distances of 3.2–3.5 mm. In comparison, Liu et al. (2025) reported average symmetric surface distance from 1.4 to 2.1 mm for most models [21]. This improvement is especially significant given the anatomical challenges of the rectal structure, including its variable content, shape, and proximity to other pelvic organs. The consistent performance across various disease types suggests our model learned robust, generalized features of rectal anatomy rather than memorized patterns specific to prostate or cervical cancer.

Although the auxiliary sex prediction task is not for clinical deployment, it does show that 94.6% accuracy, 93.5% sensitivity, and 95.7% specificity, with an AUC of 0.98, can be achieved from pelvic CT alone. The area under the ROC curve reached 0.98, which reflects discriminatory power. To ensure robustness, confidence intervals for each metric were calculated using 5-fold cross-validation. This therefore suggests that deep networks learn anatomical correlates of sex, but this finding remains exploratory, and should not be taken to imply biological inference or direct clinical use. Previous studies, such as Gichoya et al. (2022), have demonstrated that deep learning models can discriminate demographic attributes such as race in imaging [29]. The results here may reflect a similar underlying phenomenon.

Clinically, the validation results confirm effective use. Expert radiation oncologists rated 89.2% of AI-generated rectal contours as “Excellent,” 9.1% “Fair,” and 1.7% “Poor,” with Fleiss’ kappa of 0.87 (95% CI 0.82–0.91), indicating strong inter-observer agreement [4]. Moreover, the automation reduced the average contouring time from 12.7 to ~4.3 min, which could enhance the planning efficiency in resource-limited settings. These improvements are in keeping with previous results: Lempart et al. reported a ~70% time reduction for pelvic OARs, including rectum [8]. DeSilvio et al. (2023) reported that their segmentation pipeline cut manual annotation time by 78%, even though expert confirmation still takes 5–7 min per case [30].

However, several limitations must be acknowledged. First, the single-center retrospective design has limited generalizability, and the model has not been externally validated. Second, extreme anatomical variants-for example, prior pelvic surgery or congenital anomalies-were excluded from our cohort and may challenge robustness. Third, despite strong performance metrics, the risk of overfitting cannot be fully excluded without multi-institutional testing. Fourth, we have not directly compared performance with commercial auto-contouring tools, which may have pragmatic differences in performance and usability. Fifth, the sex prediction task raises ethical considerations, particularly surrounding privacy, incidental findings, and governance; these issues will require careful consideration in further work [31]. Despite these limitations, the study provides a solid foundation for future research and clinical translation. Looking ahead, the model’s capacity for detecting subtle anatomical details offers potential for predicting radiation toxicity effects or customizing treatment plans based on anatomical features, or serving as a quality assurance tool to identify contouring errors or anatomic abnormalities that could affect planning. Multi-institutional clinical validation studies will be necessary to ensure widespread applicability; however, integrating it into treatment planning systems will provide practical benefits.

## 6. Conclusions

This study shows that deep learning can simultaneously achieve accurate rectal segmentation and surprising sex prediction ability on pelvic CT images. The model’s consistent performance across different patient cohorts, clinical acceptance, and gains in computational efficiency underscore its potential as a valuable tool in modern radiotherapy planning. As oncology continues to move toward more personalized care, integrating such AI-driven approaches may enhance both the precision and accessibility of treatment planning.

## Figures and Tables

**Table 1:** Performance comparison of different models in pelvic organ segmentation.

References	Model Architecture	Data	Evaluation Metric	Key Results
[6]	U-Net (Base Architecture)	Biomedical Images (limited data)	-	Introduced the foundational U-Net architecture, becoming a baseline for many subsequent models.
[7]	3D U-Net	Male Pelvic CT Images	DSC	DSC: 0.94 (mean) for various organs
[8]	Pelvic U-Net (Deeply supervised shuffle attention CNN)	Pelvic CT of Anal Cancer Patients and OARs	DSC & Time Savings	DSC: 0.87–0.97 Time: ~40 min (manual) → ~12 min (with correction) → ~4 min (fully automatic)
[9]	SegResNet (VAE)	Pelvic CT for OARs	DSC	DSC: 0.86 (achieved with optimized loss function)
[10]	Deep Learning-based (Patient-specific nnU-Net)	Prostate CT for Radiation Therapy	DSC	0.83 ± 0.04
[11]	Review (Various State-of-the-Art Models)	Pelvic Cancers (CT/MRI)	-	Review Article: Discusses current state-of-the-art approaches and challenges in the field.
[22]	Deep Learning-based Segmentation Model	Cervical CT for Radiation Therapy	DSC	between 0.82 and 0.87
[12]	Deep Learning-based Segmentation Model	Abdominal CT for Adiposity	-	Identified gender-specific adiposity subtypes from the segmentation data.
[19]	CNN-based architecture	prostate and rectal segmentation on CT images	DSC	DSC of over 0.69
[21]	tested 44 segmentation models	-	DSC	DSC: between 0.82 and 0.87 transformer-based models scoring higher than 0.88

Note: DSC: Dice Similarity Coefficient, OAR: Organs at Risk, MRI: Magnetic Resonance Imaging, CT: Computer tomography.

**Table 2:** Demographic and clinical characteristics of the study population.

Characteristic	Prostate Cancer (*n* = 97)	Cervical Cancer (*n* = 89)
Age (years)	68.0 ± 6.0	52.0 ± 6.5
Male, *n* (%)	97 (100)	-
Female, *n* (%)	-	89 (100)
BMI (kg/m^2^)	26.8 ± 2.5	25.4 ± 2.5
CT Slice Thickness (mm)	3.0	3.0
Rectal Volume (cm^3^)	98.4 ± 14.3	86.3 ± 13.2

**Table 3:** Detailed performance metrics for the sex prediction task.

Metric	Value (%)	95% Confidence Interval
Overall Accuracy	94.6	90.2–97.1
Sensitivity (Male)	93.5	87.9–96.6
Specificity (Female)	95.7	90.8–98.1
Positive Predictive Value	95.3	90.4–97.8
Negative Predictive Value	94.0	89.3–96.8
F1-Score	94.4	90.6–96.7

**Table 4:** Summary of segmentation performance metrics for rectal contouring.

Metric	Prostate Cancer (*n* = 97)	Cervical Cancer (*n* = 89)	*p*-Value
Dice Similarity Coefficient (DSC)	0.91 (0.88–0.93)	0.89 (0.86–0.92)	0.12
Hausdorff Distance (HD, mm)	3.2 (2.5–4.1)	3.5 (2.8–4.3)	0.08
Average Surface Distance (ASD, mm)	1.1 (0.8–1.4)	1.2 (0.9–1.5)	0.15
Precision	0.92 (0.89–0.94)	0.90 (0.87–0.93)	0.09
Recall	0.91 (0.88–0.93)	0.89 (0.86–0.92)	0.11

## Data Availability

The data presented in this study are available on request from the corresponding author.
